# Acknowledgment to Reviewers of *Insects* in 2020

**DOI:** 10.3390/insects12020094

**Published:** 2021-01-22

**Authors:** Insects Editorial Office

**Affiliations:** MDPI AG, St. Alban-Anlage 66, 4052 Basel, Switzerland

Peer review is the driving force of journal development, and reviewers are gatekeepers who ensure that *Insects* maintains its standards for the high quality of its published papers. Thanks to the cooperation of our reviewers, in 2020, the median time to first decision was 15 days and the median time to publication was 36 days. The editors would like to express their sincere gratitude to the following reviewers for their precious time and dedication, regardless of whether the papers were finally published:
Aak, AndersLee, Joon HoAardema, Matthew L.Lee, Kwang-ZinAbé, HiroshiLee, Kyeong-YeollAbney, MarkLee, Kyu-ShikAbraham, CheriLee, YoosookAbro, Ghulam HussainLees, Rosemary SusanAcosta Gutiérrez, RoxanaLegalov, AndreiAdalbert, BalogLegalov, Andrey AlexandrovichAdam, PaulLehmann, Fritz OlafAdamidis, George C.Lehnert, Matthew S.Adams, ChrisLemic, DarijaAdams, ChristopherLeppla, Norman C.Adams, RachelleLessard, Bryan D.Adamski, ZbigniewLester, Philip JohnAdán, ÁngelesLewis, Leslie C.Addesso, KarlaLi, JianhongAdler, LynnLi, TengAebi, AlexLi, XuankunAhmed, Muhammad Z.Liang, WanwanAhmed, SaveerLiao, Ling-HsiuAi, XiaLiburd, Oscar E.Aikawa, TakuyaLiechti, George W.Airs, PaulLilja, TobiasAkhtar, YasminLimonta, LidiaAkihiro, NakamuraLing, ErjunAkimoto, Shin-ichiLinhari Rodrigues Pietro, Rosemeire CristinaAkino, ToshiharuLioy, SimoneAlborn, HansLis, Hab. Jerzy A.Alburaki, MohamedLittle, Nathan S.Alevi, Kaio Cesar ChaboliLiu, HoupingAlexander, James E.Liu, WenAlhmedi, AmmarLlyasov, Rustem A.Allan, Sandra A.Lo Giudice, AngelinaAllen, Kerry ClintLo Verde, GabriellaAlugubelly, NavathaLognay, Georges C.Aluja, MartínLoni, AugustoAlves E. Silva, Thiago LuizLooney, ChrisAlves, Tavvs M.Lorenzen, MarceAlyokhin, AndreiLoreto, Elgion LAmarasekare, Kaushalya G.Lövei, GáborAmeixa, Olga M.C.C.Lowenstein, David M.Amiri, EsmaeilLowry, KymAmsalem, EtyaLu, Hsiao-lingAnastasaki, EiriniLu, ZhaozhiAnaya-López, Jose LuisLuan, Yun-XiaAnderbrant, OlleLucas, JaneAndersen, Jeremy C.Luckhart, ShirleyAndo, IstvanLudwiczuk, AgnieszkaAndongma, Awawing AnjwengwoLühken, RenkeAndonov, SretenLuke, SarahAndreadis, StefanosŁukowski, AdrianAndrejko, MariolaLuo, BinAndrés, PilarLuo, Kai-JunAngelella, GinaLupi, DanielaAngelini, David R.Lycett, Gareth J.Antonatos, SpyridonLyon, LaurenAntonets, KirillMa, YunApperson, Charles S.MacLean, DavidArab, AlbertoMacVean, Charles M.Arango, Rachel A.Madeira, FilipeArbea, JavierMaekawa, KiyotoArce, Carla MarquesMaeno, Koutaro OuldArias, Elizabeth T.Maeto, KaoruAristizábal, Luis F.Maggi, FilippoArmbruster, PeterMagoga, GiuliaArnal Barbedo, Jayme GarciaMagori, KrisztianArnao, Marino BañónMaicas, SergiArthur, FrankMaiega, HamidouAscrizzi, RobertaMainali, Bishwo PrasadAshbrook, Aaron R.Maisonhaute, Julie-ÉléonoreAshfaq, MuhammadMaistrello, LaraAshour, Mohamed-BassemMaixner, MichaelAtanasova, Daniela I.Malacrinò, AntoninoAthanasios, DalakourasMalaquias, José BrunoAthanassiou, Christos G.Malcolm, StephenAthey, KacieMalenovský, IgorAtkinson, ThomasMalrey, LeeAttardo, Geoffrey M.Mammola, StefanoAuad, Alexander M.Mandrioli, MauroAudsley, NeilManicardi, Gian CarloAugustinos, AntoniosManino, AuloAugustinos, Antonios A.Mankin, Richard W.Aulicky, RadekMann, Rajinder S.Austel, NadineMannu, RobertoAvalos, ArianMans, BenAvanesyan, AlinaMansour, RamziAvarguès-Weber, AuroreMäntylä, ElinaAvilla, JesúsMantzoukas, SpiridonAvtzis, Dimitrios N.Mao, MengAyvaz, AbdurrahmanMarcantonio, MatteoBackus, ElaineMargaritopoulos, John T.Bae, Mi-JungMarin Gomez, Oscar H.Baer-Imhoof, BarbaraMarini, GiovanniBagnoli, BrunoMarrelli, MauroBahar, Md HabibullahMartemyanov, ViatcheslavBahder, BrianMartin, ArnaudBaiocchi, TiffanyMartín, FernandoBakkali, MohammedMartin, StephenBakonyi, GáborMartín-Blázquez, RubénBakovic, VidMartínez, Luis CarlosBalakirev, Evgeniy S.Martínez-Guitarte, José-luisBálint, JánosMartino, Maria ElenaBaquero Martin, EnriqueMartins, GustavoBara, JeffreyMartín-Vega, DanielBaracchi, DavidMartoni, FrancescoBarbercheck, MaryMarullo, RitaBarbero, FrancescaMarzachí, CristinaBarbosa, BrunoMas, FloreBarceló, CarlosMasetti, AntonioBarić, BoženaMassa, BrunoBarker, Gary M.Massimino Cocuzza, Giuseppe ErosBarman, Apurba K.Mastore, MaristellaBarra-Bucarei, LorenaMastrantonio, ValentinaBarrera, JuanMateos, EduardoBarrera, RobertoMathias, DerrickBartholomay, LyricMathiopoulos, KostasBartlett, CharlesMatošević, DinkaBaser, NurayMatsumoto, YasuhikoBasnet, SanjayMatthews, Benjamin J.Battaglia, DonatellaMay, Michael L.Battiston, RobertoMayo, PeterBatuman, OzgurMazo-Vargas, AnyiBawin, ThomasMazur, MiloszBaxter, Simon WadeMazza, GiuseppeBeaurepaire, AlexisMazzi, DominiqueBecchimanzi, AndreaMazzon, LucaBecher, Paul G.Mazzoni, EmanueleBeebe, NigelMazzoni, ValerioBelcari, AntonioMcAfee, AlisonBelk, MarkMcCulloch, Graham A.Belles, XavierMcCullough, Erin L.Bellini, RoméoMcEvoy, Peter B.Belluco, SimoneMcGhee, PeterBenecke, MarkMcKirdy, SimonBenedict, Mark Q.McMillan, Joseph R.Benelli, GiovanniMcNeil, JeremyBenhadi-Marín, JacintoMeagher, Robert L.Benoit, Joshua B.Meccariello, AngelaBenowitz, Kyle M.Medley, Kim A.Benrey, BettyMedo, JurajBento, José Maurício SimõesMeikle, WilliamBeresford, DavidMeiklejohn, Kelly A.Bernaola, LinaMeiners, Joan M.Bernard, Ernest C.Melendez, JesykaBernard, RafałMenna-Barreto, Rubem Figueiredo SadokBernotienė, RasaMenocal, Octavio A.Berson, JacobMent, DanaBertin, SabrinaMenuz, KarenBessin, RicMenze, Michael A.Bessin, RicardoMerritt, DavidBeugnet, FredericMesselink, GerbenBhadriraju, SubramanyamMetz, Bradley N.Bienefeld, KasparMeuti, MeganBieńkowska, MałgorzataMichaelakis, AntoniosBilleter, Jean-ChristopheMichalik, AnnaBird, Lisa JaneMihai-Leonard, DudumanBistline-East, AllisonMikac, KatarinaBlackman, Roger L.Milbrath, Meghan O.GradyBlacquière, TjeerdMillar, Jocelyn G.Blanda, ValeriaMiller, Daniel R.Blanke, AlexanderMiller, JonBlatt, Suzanne E.Mills, Mary KatherineBlock, AnnaMilosavljević, IvanBobadilha, Gabrielly S.Miloshev, GeorgeBocak, LadislavMishra, DushyantBodino, NicolaMitrovic, Milana S.Bogusch, PetrMitrus, SławomirBohinc, TanjaMitsuhashi, WataruBohman, BjörnMittapelly, PriyankaBoieiro, MárioMiura, TakeshiBomphrey, RichardMiyatake, TakahisaBonacci, Emeritus OgnjenMizell, Russell F.Bonifácio, Luís Filipe PrazeresMogren, Christina L.Bonilla-Rosso, GermánMohr, Rachel M.Bonoan, RachaelMoise, EricBonos, EleftheriosMontagna, MatteoBonsignore, Carmelo PeterMonteiro, AntóniaBorges, IsabelMontoya, PabloBorrego, Ana AriasMontoya-Lerma, JamesBorsuk, GrzegorzMoraza, MaríaBorza, TudorMorrison, Lloyd W.Borzée, AmaëlMorrison, RobBoscardin, JardelMorrison, William R.Bosch, DolorsMorrone, JuanBosco, DomenicoMorrow, Jennifer L.Bosio, ChrisMorse, Douglass H.Bosso, LucianoMorse, GeoffBoto, TamaraMoruzzo, RobertaBouchard, WillMota-Sanchez, DavidBoucias, Drion G.Mottern, Jason L.Bovera, FulviaMoulton, John KevinBovo, SamueleMound, Laurence A.Brabec, DanMoyano, FranciscoBradshaw, Terence L.Moya-Raygoza, GustavoBraks, MarietaMozuraitis, RaimondasBranca, AntoineMüller, CarolineBrandmayr, PietroMüller, ChristinaBrascamp, Evert WillemMuniappan, RangaswamyBray, Alicia M.Muona, JyrkiBreed, MichaelMurata, MikaBrelsfoard, CoreyMurray, Christopher M.Brent, ColinMusolin, Dmitry L.Brettell, Laura E.Musseau, CamilleBrewer, GaryMussury, Rosilda M.Brewer, Michael J.Mutebi, John PaulBriem, FelixMutinelli, FrancoBrodie, Bekka S.Muzzi, MaurizioBrown, Brian V.Nadolny, RobynBrown, SebeNagata, ShinjiBrown, William D.Nai, Yu-ShinBrozek, JolantaNakai, MadokaBrückner, AdrianNakamori, TaizoBrunet, JohanneNaranjo, StevenBruninga-Socolar, BethanneNardi, FrancescoBryant, BartNash, Michael A.Bryantsev, AntonNava, Dori EdsonBrygadyrenko, Viktor V.Navarro, ShlomoBucek, AlesNavas González, Francisco JavierBucekova, MarcelaNchu, FelixBuczyński, PawełNedvěd, OldřichBuellesbach, JanNegron, JoseBuitenhuis, RosemarijeNelson, Michael FranceBurdfield-Steel, Emily R.Neoh, Kok-BoonBurian, Steven K.Nermut, JiříBurritt, James B.Neumayer, JohannByers, KelseyNiederegger, SentaByrd, Jason H.Nielsen, AnneCabello-Garcia, T.Nieri, RacheleCabirol, AmélieNikolaus, SzucsichCaceres, CarlosNishiwaki, HisashiCai, ChenyangNobre, Tânia MesquitaCai, MinminNofemela, Robert S.Calatayud, Paul-andréNonacs, PeterCaldara, RobertoNoriyuki, SuzukiCaleca, VirgilioNormark, BenjaminCalles-Torrez, VeronicaNorris, Edmund J.Camargo, Roberto Da SilvaNovak, LajosCameron, Stephen L.Novgorodova, Tatiana A.Cammack, Jonathan A.Ntalli, Nikoletta G.Campbell, Corey L.Nugnes, FrancescoCampbell, EwanNunes, Francis Morais FrancoCampos, MateusNyabuga, FranklinCaparros Megido, RudyNzelu, ChukwunonsoCapbell, BrittanyO’Neal, ScottCappa, FedericoO’neill, KevinCappellozza, SilviaObrycki, John J.Caprio, MichaelOchoa, RonaldoCarapelli, AntonioOdemer, RichardCardé, Ring T.Okamoto, NaokiCardoso, Danon ClemesOlafson, PiaCardoso, PedroOleksa, AndrzejCargnus, ElenaOliveira, Maria ManuelCarlton, ChristopherOliveira-Júnior, José Max Barbosa DeCarmona-Galindo, VictorOliver, JonathanCarvalho, Danilo OliveiraOliver, ShünéCarvalho, Maria OtiliaOlmeda, Ángeles SoniaCasalla Daza, RobinOlmeda, SoniaCastane, CristinaOlson, KennethCastaño-Meneses, GabrielaOno, HajimeCastrejón-Gómez, Víctor RogelioOnufrieva, Ksenia S.Castro, Mariana S.Oppenheim, SaraCaterino, MichaelOrlova-Bienkowskaja, Marina J.Cavalieri, VincenzoOromí, PedroCeccherini, Maria TeresaOrtiz, AntonioCerini, FrancescoOsawa, NaoyaCeryngier, PiotrOsborn, RachelCespedes-Acuna, Carlos L.Osborne, Lance S.Chakrabarti, PriyadarshiniOsbrink, WesteChandler, DaveOsório, HugoChandran, RakkiyappanOstiguy, NancyChandrasekaran, MurugesanOtaki, JojiChang, Chu SikOtero-Bravo, AlejandroChapman, NadineOtti, OliverChappell, Thomas M.Otuka, AkiraCharbonneau, DanielOumbouke, WelbeckCharriere, Jean-DanielOxford, GeoffChartier, MarionOzaki, KatsuhisaChaves, Luis FernandoPaine, TimothyChen, ChaoPaiva, Patrícia Maria GuedesChen, JianwuPalacios Vargas, José GuadalupeChen, KepingPallarés, SusanaChen, LiPandey, Atul KumarChen, Xi’enPandey, GunjanChen, XiaoPapanikolaou, Nikos E.Chen, Xiao-ShengPape, ThomasChen, XuedongParadkar, PrasadChen, Yanping (Judy)Park, Hyun-WooCheng, Sen-SungPark, Il-KwonCheng, Xiao-WenPark, Kye ChungChernyak, SergeiPark, Soo JeanChesnais, QuentinPark, YoonseongChevalier, MathieuParmentier, ThomasChieco, CamillaParry, HazelChitarrini, GiuliaParys, KatherineChłond, DominikPascucci, IlariaChoi, Sei-WoongPaskewitz, SusanChoi, Won IlPatil, Chandrashekhar DevidasChong, Juang-HorngPaul, AmanChowański, SzymonPaulson, DennisChristofferson, RebeccaPavan, FrancescoChroni, AntoniaPavela, RomanChuang, Ting-WuPaxton, RobertChuang, Wen-PoPearson, DavidCianferoni, FabioPeck, MichaelCini, AlessandroPedro, NavesClaborn, DavidPeluso, IlariaClark, Nicholas J.Peñaflor, Maria FernandaClavijo-McCormick, AndreaPeper, Steven TClifton, Eric HaroldPereira, AdrianoClissold, FionaPereira, Eliseu José GuedesCloyd, RaymondPereira-Peixoto, Maria-HelenaCoates, BradPérez-López, EdelCocco, ArturoPérez-Marcos, MaríaCognato, AnthonyPérez-Martínez, SandraColacci, MarcoPeri, EzioColetta Filho, Helvecio DellaPerito, BrunellaColgan, Thomas JPerkin, Lindsey CCollatz, JanaPernek, MilanCollier, RosemaryPessoa, Luis Gustavo AmorimCollins, William R.Peterson, Brittany F.Colonnelli, EnzoPfeiffer, DouglasConcepción Gutierrez, AlejandraPfeiler, EdwardCondamine, Fabien L.Phillips, Thomas W.Conlon, Benjamin H.Phocas, FlorenceCônsoli, Fernando LuísPicimbon, Jean-FrançoisConti, BarbaraPick, LeslieCook, David F.Pickett, Charles H.Cook, StephenPienaar, RonelCook, Steven C.Piersanti, SilvanaCoombes, CandicePietri, JoseCoon, KerriPilz, ChristinaCoop, LeonardPimentel, Ida ChapavalCooper, AnastasiaPintar, Matthew R.Cooper, PaulPirk, Christian W.W.Cooper, W. RodneyPita, SebastianCooper, William RodneyPitts-Singer, TheresaCopren, Kirsten A.Pizzolotto, RobertoCorcoran, JacobPizzorno, Marie C.Cordero-Rivera, AdolfoPlarre, RuedigerCork, SusanPleasants, John M.Corona, MiguelPleydell, David R.J.Correa, MaríaPodsiadlowski, LarsCorrias, FrancescoPoidatz, JulietteCossu, PieroPoinar, GeorgeCosta-Da-Silva, André LuisPolaszek, AndrewCostello, Michael J.Polidori, CarloCottrell, Ted E.Polilov, Alexey A.Crava, Cristina M.Pollice, AlessandraCremer, SylviaPondeville, EmilieCristofaro, MassimoPons, XavierCrossley, Michael S.Pope, TomCrumiere, Antonin J.J.Popham, Holly J.Csóka, GyörgyPorcel, MarioCuevas, JuliánPorcelli, FrancescoCuli, JoaquimPorporato, MarcoCulliney, Thomas W.Porretta, DanieleCuthbertson, Andrew G. S.Port, GordonCzachorowski, StanisławPortilla, Maribel R.Czaczkes, TomerPorto, André Luiz MeleiroCzarniewska, ElżbietaPorto, Morgana Maria FonsecaCzerniewicz, PawełPotekhin, AlexeyD’Alessandro, PaolaPotter, DanielDa Cunha, Hélida FerreiraPovelones, MikeDa Silva Motta, Erick VicentePozzebon, AlbertoDa Silva Torres, Christian Sherley AraujoPrager, SeanDa Silva, PedroPramod, SreepriyaDacher, MatthieuPreisser, EvanDaferera, DimitraPrendin, AngelaDai, Shu-MeiPrešern, JanezDajoz, IsabellePusceddu, MichelinaDamos, Petros T.Quarrell, StephenDani, Francesca RomanaQuerner, PascalDaniels, JaretQuesada-Moraga, EnriqueDany, LionelQuinonez, Fausto ValenzuelaDara, Surendra K.Quintanilla, MarisolDarvas, BélaRadek, RenateDavidson, Melanie MillicentRajabi, HamedDavis, JeffRajamohan, ArunDe Almeida, Lucia MassuttiRajarapu, Swapna PriyaDe Barros, AntonioRajeswaran, JagadeesanDe Bellis, LuigiRakauskas, RimantasDe Boni, AnnalisaRamadan, Mohsen M.De Clercq, PatrickRamalho, ManuelaDe Cristofaro, AntonioRamsey, Samuel D.De Freitas Bueno, Regiane Cristina OliveiraRand, Tatyana A.De Freitas Soares, Filippe EliasRanger, Christopher MDe Marchi, Bruno RossittoRashed, ArashDe Menezes, Cristiano RagagninRašić, GordanaDe Moura, Ana PintoRassati, DavideDe Oliveira, Eugênio EduardoRaupach, Michael J.De Rojas, ManuelRaymann, KasieDe Souza Loureiro, ElisângelaRaymond, BenDe Souza Trindade, GilianeRebora, ManuelaDe Souza, Danival JoséReding, MichaelDearden, PeterReeves, Lawrence E.Deboutte, WardRehman, Junaid UrDeisig, NinaReich, IngaDel Pozo-Valdivia, AlejandroRemelli, SaraDelalibera, ÍtaloRemesar, SusanaDelaunay, PascalRenkema, JustinDella Torre, AlessandraRenou, MichelDelsinne, ThibautRenoz, FrançoisDenecke, ShaneRevadi, SantoshDepalo, LauraReznik, Sergey YaDerek, WollerRibani, AnisaDerkarabetian, ShahanRibeiro, Bergmann MoraisDes Marteaux, LaurenRicigliano, Vincent A.Despland, EmmaRiddick, Eric WellingtonDevillers, JamesRiddiford, Lynn M.Dezmirean, Daniel S.Ridout, Mary E.Di Pasquale, GaranceRiedel, AlexDias, GuilhermeRiehle, MichaelDias, Vanessa SimõesRiesch, RudigerDíaz-Fleischer, FranciscoRijal, JhalendraDietrich, Christopher H.Riley, DavidDiez, YagoRiley, Edward G.Dillman, AdlerRinkevich, FrankDimitratos, Spiros D.Riudavets, JordiDindo, Maria LuisaRivera-Pérez, CrisalejandraDisney, HenryRivera-Vega, LorenDitrich, TomášRizzo, RobertoDittmer, JessicaRoberts, JoeDively, GalenRoberts, JohnDixit, SameerRobinson, KarlDmitryjuk, MałgorzataRobinson, Willard S.Do Amaral Melo, Ana ClaudiaRobledo Quintos, Norma RDobeš, PavelRobuchon, MarineDobson, Adam J.Rodriguero, MarcelaDolan, BenjaminRodrigues Santiago, LeandroDombrovsky, AvivRodrigues, AndreDomingue, MichaelRodrigues, SérgioDomínguez-Ramírez, Julio LeninRodriguez Arrieta, JuanitaDonald, Claire L.Rodríguez González, ÁlvaroDong, KeRodríguez, Armando AlexeiDonkersley, PhilipRodriguez, PatriciaDonly, CamRodriguez, RafaelDonnell, David M.Rodriguez-Andres, JulioDoremus, MatthewRoe, AmandaDorin, AlanRoe, Richard MichaelDos Santos Niculau, EdenilsonRoeder, KarlDouglas, HumeRogers, MaryDouglas, Hume B.Roggero, AngelaDowse, HaroldRohlfs, MarkoDražić, MajaRojas, Julio C.Drees, ClaudiaRojas, SandraDrucker, MartinRomán López, PabloDrummond, Frank Francis A.Romanowski, JerzyDu, Yu-ZhouRömer, DanielaDu, ZhentingRondoni, GabrieleDuan, JianRönkä, Katja HelenaDuarte, AmilcarRoques, AlainDudley, Kevin J.Rosa, CristinaDufour, Bernard P.Rosell, Rosemarie C.Duplouy, AnneRosendale, Andrew J.Dupuis, Julian R.Rosengaus, RebecaDurak, RomaRosenkranz, PeterDutra, HevertonRoss, PerranDutto, MorenoRossi, ElisabettaDuvic, BernardRossi, MarikaDyson, Paul J.Rostislav, ZemekDzida, KatarzynaRothman, Jason A.Eacock, AmyRougeot, JulienEady, PaulRouttu, JarkkoEbadollahi, AsgarRowland, KimberlyEben, AstridRowley, Daniel L.Ebert, Timothy A.Różalska, SylwiaEdwards, MartinRozman, VlatkaEhrlich, HermannRuberson, JohnElek, ZoltánRubio, LuisEL-Hefny, MervatRubiolo, PatriziaEliash, NuritRuchin, Alexander B.Eliopoulos, Panagiotis A.Rueppell, OlavElliot, Simon LukeRuiu, LucaElsensohn, Johanna E.Rumbos, Christos I.El-Wakeil, NabilRust, MichaelEmami, NoushinRuther, JoachimEndersby, NancyRyabov, EugeneEngl, TobiasSabbatini Peverieri, GiuseppinoEngström, YlvaSadd, BenjaminEnnos, RolandSagili, RameshEntling, WiebkeSagona, SimonaErban, TomasSaigusa, KiyoshiEspino, LuisSaito, KazuyaEsquivel, BaldomeroSakashita, TetsuyaEtzler, Frank E.Salom, ScottEveillard, SandrineSaloña-Bordas, Marta I.Evenhuis, Neal LuitSalvemini, MarcoEvergetis, EpameinondasSambaraju, KishanEyer, Pierre-AndreSamu, FerencEyres, IsobelSamuels, Richard IanFalabella, PatriziaSamy, AbdallahFallon, Ann M.Sanborn, Allen FFan, LushengSánchez Peña, SergioFanti, PaoloSandrelli, FedericaFaraone, NicolettaSantos, Dulce C.N.Fardisi, Mahsa N.Santos-Garcia, DiegoFarías Franco, FernandoSatta, AlbertoFekih, Ibtissem BenSauka, DiegoFeldhaar, HeikeSaul-Gershenz, Leslie S.Feldmeyer, BarbaraSavoldelli, SaraFeng, HonglinSbordoni, ValerioFeresin, GabrielaScharf, MichaelFerracini, ChiaraScheffrahn, Rudolf H.Ferrante, MarcoSchell, Scott P.Ferriol, InmaculadaSchlesinger, Matthew D.Ferveur, Jean-FrancoisSchmidt, Jason M.Fescemyer, HowardSchmidt, Thomas L.Fettig, ChrisSchmitt, EricFiedler, KonradSchmitt, MichaelFields, PaulSchneider, ClémentFigueroa, LauraSchomann, Andrea M.Fikáček, MartinSchrallhammer, MartinaFilipiak, MichałSciarretta, AndreaFirlej, AnnabelleScortichini, MarcoFlaherty, Leah E.Scott, IanFlamini, GuidoScott, Maxwell J.Fleming, Daniel E.Scully, ErinFlórez, Laura V.Seeger, MarkFLORIS, IgnazioSegers, FranciscaFlynt, Alex S.Segoli, MichalFois, MauroSeimandi-Corda, GaëtanFoissac, XavierSeo, ShigemiFöldvári, GáborSeppa, PerttuFollett, Peter A.Sequeira, Andrea S.Foray, VincentSequeira, Richard V.Forister, Matthew LewisSerrano, JoseForsgren, EvaSerraõ, José ÉduardoForshage, MattiasSeshadri, ArathiFoster, ChrisSethuraman, ArunFoster, Leonard J.Severin, FrancescoFraile, Paula GarciaSeverson, David W.França, TâmaraSevilla-Morán, BeatrizFrancati, SantoloSexton, Nicole R.Francikowski, JacekSforza, RenéFranco, José CarlosSgolastra, FabioFrank, Erik T.Shapiro Ilan, DavidFrank, KevinShapiro, ArthurFraser, JohannaSharaf, AbdoallahFreitas-Astúa, JulianaSharakhov, IgorFritz, Megan L.Sharakhova, MariaFrizzi, FilippoSharkey, Camilla R.Fruttero, Leonardo L.Sharma, LavFu, Zhen (Daisy)Shaw, RosalindFuchs, BenjaminShelby, Kent S.Fuehrer, Hans-PeterShelomi, MatanFuentealba, AlvaroShelton, AnthonyFujiwara, HaruhikoSherwood, PatrickFurlan, LorenzoShiao, Shin-HongGábor, FöldváriShih, Hsien-TzungGabrieli, PaoloShilts, TurksenGaglianone, Maria CristinaShimmi, OsamuGajda, AnnaShin, SeunggwanGaletto, LucianaShrestha, DeepakGalko, JurajShrestha, ManiGallego Cambronero, DiegoSiegel, JoelGalli, LorisSielezniew, MarcinGamboa, MaribetSigle, LeahGanassi, SoniaSilberbush, AlonGanjisaffar, FatemehSilva, Maria FátimaGao, BeiSilva-Filho, Marcio C.Gao, CongfenSilver, KristopherGarcia Del Pino, FernandoSim, Sheina B.Garcia, Flávio Roberto MelloSimanonok, MichaelGarcía, Jair E.Simo, LadislavGarcía-Marín, RamónSimões, Zilá Luz PaulinoGarcía-Segovia, PurificaciónSimone-Finstrom, Michael D.Garganese, FrancescaSimonetto, AnnaGaronna, Antonio PietroSinclair, Bradley J.Garrido-Jurado, InmaculadaSinger, Michael C.Garvey, MichaelSingh, HarpalGautam, Sandipa GautamSingh, Kumar SaurabhGavinelli, FedericoSinotte, Veronica M.Gawne, RichardSiozios, StefanosGebiola, MarcoŠípek, PetrGeden, Christopher J.Skaldina, OksanaGemeno, CésarSkartveit, JohnGendrin, MathildeSkevington, JeffGerken, Alison R.Skorokhod, OleksiiGerth, MichaelSkouras, PanagiotisGessner, GuidoSkovgård, HenrikGherman, Calin MirceaSkuhrovec, JiříGhiringhelli, Pablo DanielSlotsbo, StineGhosh, PrarthanaSmith, Hugh A.Giatropoulos, Athanassios K.Smith, Ryan C.Gibert, Jean-MichelSmodiš Škerl, MajaGiglio, AnitaSnow, Jonathan W.Giliomee, Johannes HumanSoares, António OnofreGilligan, Todd M.Sobhy, Islam S.Ginzel, Matthew D.Sollai, GiorgiaGiovenazzo, PierreSolodovnikov, AlexeyGippet, JérômeSommaggio, DanieleGisder, SebastianSong, HojunGiulianini, Piero GiulioSoroker, VictoriaGiunti, GiuliaSosa Gomez, DanielGlare, TravisSouza, DarianeGlazer, ItamarSøvik, EirikGlowacz, AdamSpafford, HelenGoblirsch, Michael J.Sparks, MichaelGogala, MatijaSperanza, StefanoGomez Silva, JaniseteSpinelli, FrancescoGomez-Marco, FrancescSpivak, MarlaGomis-Cebolla, JoaquinSrinivasan, RajagopalbabuGondhalekar, Ameya DSrinivasan, RamasamyGonella, ElenaSrygley, Robert BaxterGonzález Hernández, HéctorSt Laurent, BrandyceGonzalez, EzequielStabentheiner, AntonGonzález-Zamora, José EnriqueStahl, Judith M.Goodman, Alan G.Stanimirovic, ZoranGoolsby, JohnStark, John D.Gordon, EricStathers, TanyaGottdenker, NicoleStauffer, ChristianGould, JuliStefano, CassanelliGrabowski, Nils Th.Steiner, Florian M.Grant, WilliamStejskal, VaclavGrassl, JuliaStelinski, Lukasz L.Grasso, Donato AntonioSterzyńska, MariaGray, AlisonStockan, JenniGreen, Delbert AndréStocker, AnnGregorc, AlešStockton, DaraGrodowitz, Michael JayStolle, EckartGrodzicki, PrzemysławStoops, CraigGrodzki, WojciechStorer, Caroline G.Gross, AaronStout, Michael J.Gross, JürgenStraub, LarsGruwell, MatthewStrauss, JohannesGrzywacz, AndrzejStreinzer, MartinGu, LiuqiStrong, Donald R.Guarino, SalvatoreStrutzenberger, PatrickGuastella, DevidStyrsky, John D.Guedes, Raul Narciso CarvalhoSu, JianyaGuédot, ChristelleSubramanyam, BhadrirajuGuerra, Patrick AnthonySugahara, RyoheiGuerra-Grenier, EricSugiura, ShinjiGuerrero, AngelSukhodolskaya, RaisaGui, ShunhuaSuma, PompeoGuichard, MatthieuSutherland, Andrew M.Guidi, AlessandraSuzuki, Masataka G.Gupta, KuldeepSuzuki, TakeshiGut, Larry J.Suzuki, YasutsuguGutierrez, AndrewSuzuki, YuichiroGyawali, NarayanSvecnjak, LidijaHaddi, KhalidSvensson, GlennHagstrum, David W.Swanson, Brook O.Halbert, Susan E.Sweeney, JonHall, David RobertSweet, Andrew D.Hall, Loyal P.Swengel, Ann B.Hall, MarkSynowiec, AgnieszkaHambäck, Peter A.Syromyatnikov, Mikhail Y.Hamerlik, LadislavSytykiewicz, HubertHanly, JoeSzentgyörgyi, HajnalkaHano, ChristopheSzmidt, Alfred EdwardHarano, Ken-ichiSzwedo, JacekHarbison, Susan T.Tabata, JunHarmon, JasonTak, JunHyungHarmon-Threatt, Alexandra N.Takamatsu, DaisukeHarris, KarenTakanashi, TakumaHarrison, Mark CTakiya, ShigeharuHarten, HeikoTalley, JustinHaseeb, MuhammadTanaka, HiromitsuHaselton, Aaron T.Tanaka, KazuhiroHashimoto, YoshiakiTanaka, ToshiharuHassan, ErrolTang, Cheng-MingHata, Fernando TeruhikoTang, QingfengHatano, EduardoTang, Xiao-TianHatt, SéverinTarasco, EustachioHatteland, Bjørn ArildTarone, AaronHatting, JustinTartally, AndrásHayford, Barbara L.Tatsuke, TsuneyukiHeadrick, David H.Tavella, LucianaHeckel, David G.Taylor, David B.Heckenhauer, JacquelineTecher, Maéva AngéliqueHegde, ShivanandTedeschi, RosemarieHeil, MartinTeets, Nicholas M.Helden, MaartenTena, AlejandroHellemans, SimonTennessen, Jason M.Hemerik, LiaTerblanche, John S.Henderson, GreggTessier, Jack T.Henriksen, Marie VestergaardThayer, MargaretHenriques, DoraTheodorou, PanagiotisHernández-Martínez, PatriciaTheopold, UlrichHernández-Rodríguez, CésarThistlewood, HowardHerrera, MartaThomas, Donald B.Herrin, DavidThomas, John C.Herrmann, MatthiasThöming, GundaHertäg, CorinneThomson, LindaHerz, AnnetteThrasyvoulou, AndreasHesson, JennyTimmons, LisaHiggins, Robert J.Tinaut, AlbertoHill, TomTkaczuk, CezaryHiller, ThomasToepfer, StefanHillyer, JulianToft, SørenHimuro, ChihiroTogawa, ToruHiroyoshi, SatoshiTojo, KojiHjelmen, Carl E.Tokuda, GakuHoelmer, KimTollerup, KrisHoffmann, Klaus H.Tomanović, ŽeljkoHogsette, JerryTomaszewska, Kazimiera WiolettaHojo, Masaru K.Tonina, LorenzoHolland, JefferyToševski, IvoHolland, John M.Tougeron, KévinHolmes, Christopher J.Toyoda, HideyoshiHolst, NielsTrager, Matthew D.Holt, Scott M.Tragust, SimonHoluša, JaroslavTrdan, StanislavHonnen, Ann-ChristinTrebicki, PiotrHooks, CerrutiTrematerra, PasqualeHorgan, FinbarrTriana, OmarHorowitz, Abraham RamiTriapitsyn, Serguei VladimirovichHorsburgh, AnnTrivellone, ValeriaHorton, David R.Troczka, BartlomiejHorváth, GáborTrotta, VincenzoHoward, Daniel R.Trotter, TalbotHribar, LawrenceTrowbridge, Amy M.Hristov, Peter IvanovTrumble, John T.Hua, HongxiaTrumbo, StephenHuang, HuaguoTsagkarakis, AntoniosHuang, Jen-PanTsagkarakou, AnastasiaHuang, Rong-NanTschinkel, Walter R.Huang, YongpingTsuchida, KojiHughes, GabrielTsujimoto, HitoshiHull, J. JoeTuda, MidoriHung, Keng-Lou JamesTuno, NobukoHunter, DavidTürke, ManfredHunter, Wayne BrianTurnbull, Matthew W.Husemann, MartinTurney, ShaunHuybrechts, RogerTyukmaeva, Venera I.Huynh, ManUdaka, HirokoHyrsl, PavelUgajin, AtsushiIatsenko, IgorUgolini, AlbertoIbrahim, Sulaiman SadiUnderwood, RobynIchim, Mihael CristinUngureanu, CameliaIglesias, LindsyUno, TomohideIijima, NoriakiUrban, JulieIlinskiy, YuriyUrbaneja-Bernat, PabloIllan, JavierUrbański, ArkadiuszIlyasov, RustemUshakova, Nina A.Ingber, David A.Uspensky, IgorIngham, VictoriaVacas, SandraIngwell, Laura L.Valdez, JoseInouye, David W.Van Asch, BarbaraIoriatti, ClaudioVan Belleghem, StevenIrmler, UlrichVan Breugel, FlorisIsmail, Hanafy MahmoudVan Den Berg, JohnnieIsman, Murray B.Van Der Blom, JanItakura, ShujiVarnon, Christopher A.Ivković, MarijaVasconcelos, Ana C.O.Iwata, RyutaroVea, Isabelle MifomIzri, ArezkiVeldtman, RuanJacobus, Luke M.Velentzas, Athanassios D.Jain, Ritesh G.Venthur, HerbertJakobs-Schönwandt, DesireeVerble-Pearson, RobinJaloux, BrunoVerhulst, Eveline C.James, PeterVianna, María FlorenciaJan, ŠobotníkVieira, Paulo CézarJaneček, ŠtěpánVijendravarma, Roshan-KumarJaskuła, RadomirVilcinskas, AndreasJavan, GulnazVillet, Martin H.Jess, StephenVinauger, ClementJílková, VeronikaVirlas, Eduardo GabrielJohnson, Melissa AnneVolf, PetrJones, CarolVolkovitsh, Mark G.Jones, Susan C.Vollhardt, Ines M.G.Joseph, ShimatVuts, JozsefJoyce, AndreaWada-Katsumata, Ayako K.Jucker, CostanzaWade, Michael J.Judd, TimothyWagner, HerbertJuliano, StevenWakefield, Maureen ElizabethJung, ChuleuiWalgenbach, JamesKaczmarczyk-Ziemba, AgnieszkaWalker, ThomasKafle, LekhnathWaller, Deborah AnnKageyama, DaisukeWalters, KeithKaila, LauriWalton, WilliamKAINOH, YooichiWan, HuKajtoch, ŁukaszWang, ChenzhuKakumanu, Madhavi L.Wang, HaikouKalampalikis, NikosWang, HuabingKamiński, Marcin JanWang, Hurng-YiKandasamy, DineshkumarWang, LiandeKankare, MaariaWang, XiaoyiKaramaouna, FilitsaWang, XingengKärhä, KalleWang, YanKariyat, RupeshWare, JessicaKaufman, Phillip E.Wari, DavidKaur, RupinderWasserlauf, Irina E.Kawasaki, HidekiWassmer, ThomasKazimírová, MariaWatanabe, HidehiroKędzior, RenataWaterhouse, RobertKeena, Melody A.Waters, James S.Kehrli, PatrikWay, Michael OrrinKelly, PatrickWebb, Cameron E.Kelly, SethWebster, Thomas C.Kennedy, George G.Weeks, EmmaKerchev, IvanWeiner, Wanda MariaKevill, JessicaWeldegergis, Berhane T.Kezic, NikolaWells, Jeffrey D.Khan, AyubWerle, Christopher T.Kher, SwaroopWermelinger, BeatKikawada, TakahiroWest, Natalie M.Kim, Dong SoonWestby, KathleenKim, HyojoongWestby, KatieKim, Ki-YoungWhite, JenniferKimura, Masahito T.Whitten, Miranda M.A.King, LindaWhittington, AndrewKirichenko, NataliaWhyard, SteveKirker, Grant T.Wieczorek, KarinaKitazumi, AiWiggins, GregKline, DanielWijnveld, MichielKlooster, Wendy S.Wild, Alexander L.Klotz, Stephen A.Wilder, ShawnKlubertanz, Tom H.Wilhelm, GerthaKłyś, MałgorzataWilke, AndreKnapp, JessicaWilke, Andre Barreto BrunoKnapp, Markus M.Williams, Christopher D.Knapp, MichalWilliams, JohnKnisley, Charles BarryWilliams, TrevorKnyshov, AlexanderWilson, BlakeKobori, YouichiWilson, HoustonKoch, Jonathan B.Wilson, MichaelKodrik, DaliborWinkler, DanielKoekemoer, Lizette L.Wise, JohnKohlmeier, PhilipWitzgall, PeterKomar, NicholasWojda, Iwona E.Konagaya, TatsuroWoodcock, BenKoné, N’Golo AbdoulayeWorthen, Wade B.Konstantopoulou, MariaWosula, EverlyneKonvička, MartinWright, DenisKooij, Pepijn W.Wright, Mark G.Košťál, VladimírWu, JianingKouřimská, LenkaWu, LiweiKovac, LubomirWu, ShaohuiKovačić, MarinWu, XiaofengKoyack, MarcWybouw, NickyKozlova, OlgaWyckhuys, KrisKratochvíl, LukášXiao, YutaoKrause-Sakate, RenateXu, BaohuaKrewenka, Kristin M.Xu, GuangKrischik, Vera A.Xu, JianKrishnan, NatrajXu, JinKristan, MojcaXu, JunhuanKristensen, MichaelXu, WeiKról, NinaXue, Rui-DeKropf, MatthiasYadav, ShrutiKrueger, AndreasYamada, HananoKryukov, Vadim Y.Yanagawa, YouichiKuhar, TomYang, Chin-Cheng ScottyKulma, MartinYang, Tsung-ShiKumar, PradeepYashiro, ToshihisaKundrata, RobinYeap, Heng LinKuo, Yen-WenYee, Donald A.Kupper, MariaYee, Wee L.Kuraishi, TakayukiYen, Pei-ShiKuramitsu, KazumuYokoi, KakeruKurtti, TimothyYoshido, AtsuoKurze, ChristophYoshiga, ToyoshiKuwar, Suyog S.Yoshii, TaishiKwong, Waldan K.Yoshimura, TsuyoshiL. Cassill, DebyZach, PeterLa Corte, RoseliZahavi, TirtzaLa Spina, MichelangeloZahniser, JamesLabbé, Roselyne M.Zając, ZbigniewLaciny, AliceZanchi, CarolineLacour, Sandrine A.Zapponi, LiviaLahiri, SriyankaZarpas, KostasLaine, Roger A.Zattara, Eduardo E.Lalander, Cecilia HelenaZehner, RichardLancaster, JillZha, ChenLandaverde, PatriciaZhan, GuopingLangton-Myers, ShelleyZhang, Dan-DanLanka, SrinivasZhang, LanLaporta, Gabriel ZorelloZhang, Qing-HeLara, RickyZhang, Shi-ZeLarson, JonathanZhang, YangzhuoLarson, Nicholas R.Zhao, XinLarson, NickZheng, HaoLattorff, H. Michael G.Zhong, Guo-HuaLawson, Sarah PageZhu, Li (Licky)Laznik, ŽigaZiganshin, AyratLazzari, ClaudioZizzari, Zaira ValentinaLe Goff, GaelleZogli, Prince KudjoeLeague, GarrettZografou, KonstantinaLeather, Simon RobertZolnerowich, GregoryLebrun, Edward GZolnik, Christine P.Leclerque, AndreasZurita, AntonioLecoq, MichelZygadlo, JulioLee, Chow-YangŻyła, DagmaraLee, Jana


